# ^18^F-Fluorodeoxyglucose Positron Emission Tomography/Computed Tomography-Positive Lymph Node Endometriosis Masquerading as Lymph Node Metastasis of a Malignant Tumor

**DOI:** 10.1155/2014/648485

**Published:** 2014-08-10

**Authors:** Makoto Akiyama, Izumi Suganuma, Taisuke Mori, Izumi Kusuki, Haruo Kuroboshi, Fumitake Ito, Hiroshi Matsushima, Morio Sawada, Jo Kitawaki

**Affiliations:** Department of Obstetrics and Gynecology, Graduate School of Medical Science, Kyoto Prefectural University of Medicine, 465 Kajii-cho, Kamigyo-ku, Kyoto 602-8566, Japan

## Abstract

Endometriosis is defined as the presence of endometrium-like tissues at extrauterine sites, most commonly in the abdominal cavity. Lymph node endometriosis is a rare but clinically important type of endometriosis that can mimic lymph node metastasis of a malignant tumor. ^18^F-fluorodeoxyglucose (^18^F-FDG) positron emission tomography/computed tomography (PET/CT) is a useful tool for diagnosing malignant tumors, although it occasionally shows false positive results in tissues with high metabolic activity caused by severe inflammation. In the present report, we describe a case of lymph node endometriosis that mimicked lymph node metastasis of a malignant tumor and showed a positive result on ^18^F-FDG PET/CT. The findings of the present case suggest that lymph node endometriosis could present as swollen lymph nodes with ^18^F-FDG PET/CT-positive results and provide important information for determining an appropriate treatment strategy.

## 1. Introduction

Endometriosis—a common condition affecting 5–10% of women of reproductive age—is defined as the presence of endometrium-like tissues at extrauterine sites. The common sites of endometriosis are the pelvic peritoneum and ovaries, whereas endometriosis in extra-abdominal cavity sites, such as the respiratory or urinary organs, is rare. One theory posits that, during menstruation, endometrial debris exits the uterus through the fallopian tubes, attaches to the peritoneal surface, and proceeds to invade the tissue [1, 2]. Clinical observations indicate that endometriosis has certain metastatic characteristics, in terms of its spread through blood or lymph ducts [3–5]. Moreover, the potentially malignant characteristics of endometriosis occasionally make it difficult to identify the presence of malignancy using clinical diagnostic procedures.

Positron emission tomography (PET) is a nuclear medical imaging technique that uses radioactive material, and ^18^F-fluorodeoxyglucose (^18^F-FDG) is one of the most commonly used radionuclides to assess abnormal glucose metabolic function in tissues. Computed tomography (CT) is advantageous for the evaluation of morphological abnormalities. ^18^F-FDG PET/CT is an imaging technique that integrates ^18^F-FDG PET and CT; it enables the simultaneous evaluation of functional and morphological characteristics and contributes to the clinical diagnostic accuracy of malignancy for a variety of cancers [6–8]. However, in benign lesions that have a high glucose metabolic activity, ^18^F-FDG PET/CT often shows false positive results [9]. In the present report, we describe an initial case of ^18^F-FDG PET/CT-positive iliac lymph node endometriosis accompanied by ureter endometriosis that mimicked lymph node metastasis of a malignant tumor.

## 2. Case Presentation

**Figure 1:**
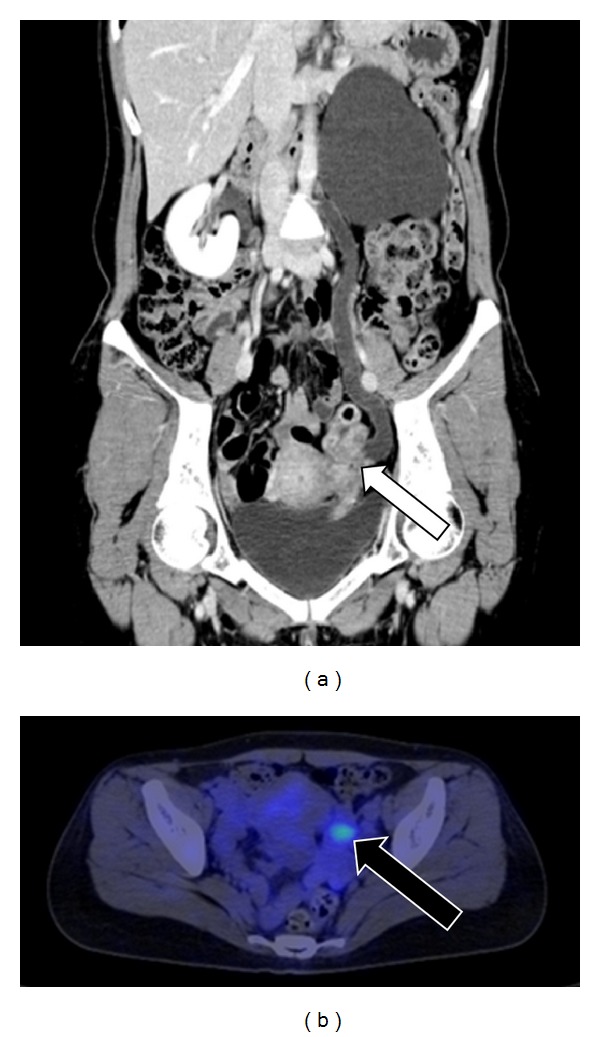
Preoperative image. (a) Coronal enhanced computed tomography (CT) showing an unclear mass that involved the left adnexa and ureter, indicated by an open arrow, along with severe left hydronephrosis and hydroureter. (b) ^18^F-fluorodeoxyglucose (^18^F-FDG) positron emission tomography/CT showing high ^18^F-FDG uptake at the internal iliac lymph node lesion, indicated by a closed arrow.

**Figure 2:**
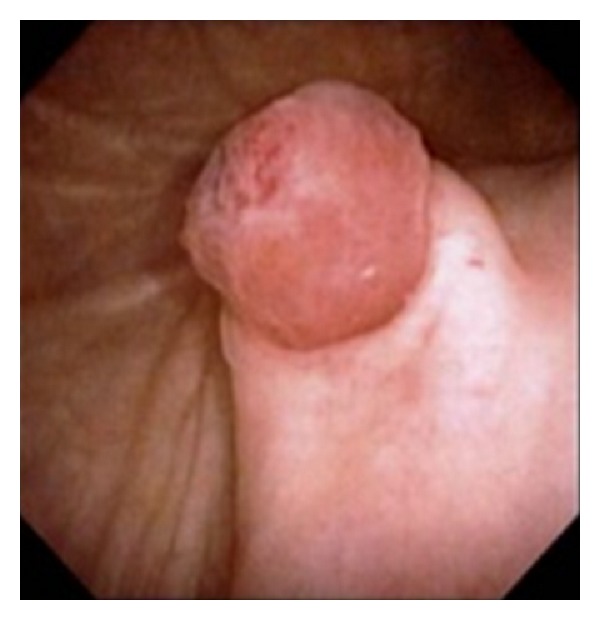
Preoperative cystoscopy findings and macroscopic appearance of a mass projecting from the left ureteral orifice to the bladder. The mass was biopsied for histological examination.

**Figure 3:**
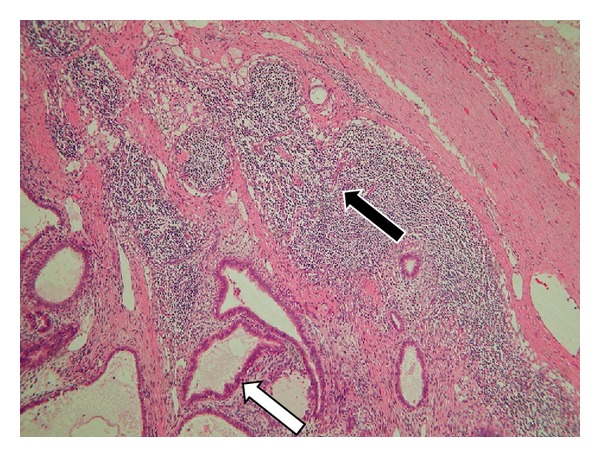
Microscopic view of the internal iliac lymph node indicating the replacement of the normal lymph node central structure with endometriotic tissues, with a residual capsule and lymphocytes at the peripheral area. The open arrow indicates an endometrium-like duct, the closed arrow indicates peripheral lymphocytes, and the area surrounded by a broken line indicates the capsule of the lymph node.

A 41-year-old woman (gravida, 2; para, 2) presented to our outpatient department with macrohematuria during her menstrual period. She had not received any hormonal treatment. No abnormal findings were noted on physical examination. Transvaginal ultrasonography indicated the presence of an unclear mass surrounding the left adnexa. The serum CA-125 level was high (251.6 IU/mL). Moreover, magnetic resonance imaging (MRI) and enhanced CT showed that the unclear mass involved the left adnexa and ureter and indicated the presence of a swollen lymph node at the left ileac artery in addition to severe left hydronephrosis and hydroureter (Figure 1(a)). Because we could not determine the origin of the tumor, we performed ^18^F-FDG PET/CT, which showed high uptake of ^18^F-FDG in the mass and iliac lymph node lesion preoperatively (Figure 1(b)). Furthermore, a subsequent cystoscopy revealed that the mass projected from the left ureteral orifice into the bladder (Figure 2). Histological evaluation of the biopsy specimen indicated the presence of nonmalignant atypical glands in the left ureter that stained positively for estrogen receptor (ER) and progesterone receptor (PR). We considered conditions such as ureteral cancer, ovarian cancer, and other benign diseases in the differential diagnosis, which was explained to the patient, along with the treatment options, risks, and benefits. Because of the possibility of malignant disease, we decided to perform total nephroureterectomy, total abdominal hysterectomy, bilateral salpingo-oophorectomy, partial omentectomy, and left swollen iliac lymph node resection. Intraoperatively, we noted a white-yellow consolidation that included the left adnexa and ureter, the left hydroureter and hydronephrosis, and a palpable left internal iliac lymph node. No other lesions were noted. The pathological diagnosis was eutopic endometrium during the secretory phase of the cycle as well as endometriosis in the left ureter, ovary, and internal iliac lymph node; no malignancy was noted. Most of the lymph node was replaced by endometriotic tissue (Figure 3), and the endometriotic lesions were found to be positive for ER, PR, and CD10 on immunohistochemical evaluation.

## 3. Discussion

To our knowledge, this is the first report of lymph node endometriosis that showed positive results on ^18^F-FDG PET/CT, indicating that clinicians need to consider the possibility of endometriosis as a cause of swollen lymph nodes with positive results on ^18^F-FDG PET/CT.

The detection of lymph node endometriosis is rare, because it is usually asymptomatic. It is rarely detected on microscopic examination of removed lymph nodes following surgery for malignant tumors. However, recent histological analyses have indicated that the lymphatic spread of endometriosis is not a rare phenomenon in women with endometriosis [3, 10]. As in the present case, endometriosis in the extra-abdominal cavity often manifests as a mass and is a known risk factor for lymph node endometriosis [11]. This complicates diagnostic procedures, which indicate the presence of a mass with swollen lymph nodes in such cases, leading clinicians to suspect the presence of a malignant tumor with lymph node metastasis.

In malignant diseases, ^18^F-FDG PET/CT is commonly used to determine the clinical stage, including the presence of metastasis or recurrence, and has high efficacy in the gynecologic field. However, several additional points should be considered when interpreting the ^18^F-FDG PET/CT findings, particularly when distinguishing between gynecologic benign and malignant tumors, in addition to the normal physiological uptake by gynecologic organs. For example, uterine leiomyoma and adenomyosis show positive uptake, ovaries and endometrium show positive results during the ovulation period, and the endometrium shows positive results during menstruation. These cases of enhanced uptake that are unrelated to malignancy make it difficult to interpret the results. Thus far, the reports of positive uptake by endometriosis are limited to endometriosis in extra-abdominal cavities, such as lung endometrioma or rapidly progressing deeply invasive endometriosis [12–14], suggesting that high glucose metabolic activity can be detected by ^18^F-FDG PET/CT only in cases of progressive endometriosis, as in the present case.

Thus, the present case provides important information for diagnosing cases of swollen lymph nodes and positive ^18^F-FDG PET/CT results, by demonstrating the potential for lymph node endometriosis to exhibit positive ^18^F-FDG PET/CT results. Therefore, clinicians should consider the possibility of lymph node endometriosis, which may aid in the determination of appropriate treatment plans, including surgical intervention, while considering the findings of exploratory laparotomy or intraoperative pathological consultation.
